# DNA methylation: a cause and consequence of type 2 diabetes

**DOI:** 10.5808/GI.2019.17.4.e38

**Published:** 2019-11-28

**Authors:** Mirang Kim

**Affiliations:** 1 Personalized Genomic Medicine Research Center, Korea Research Institute of Bioscience and Biotechnology (KRIBB), Daejeon 34141, Korea; 2 Department of Functional Genomics, University of Science and Technology (UST), Daejeon 34141, Korea

**Keywords:** diabetes, DNA methylation, epigenetics

## Abstract

DNA methylation is a relatively stable epigenetic modification that can regulate and stabilize gene expression patterns and hence establish cell identity. Because metabolic intermediates are key factors of DNA methylation and demethylation, perturbations in metabolic homeostasis can trigger alterations in cell-specific patterns of DNA methylation and contribute to disease development, including type 2 diabetes (T2D). During the past decade, genome-wide DNA methylation studies of T2D have expanded our knowledge of the molecular mechanisms underlying T2D. This review summarizes case-control studies of the DNA methylome of T2D and discusses DNA methylation as both a cause and consequence of T2D. Therefore, DNA methylation has potential as a promising T2D biomarker that can be applied to the development of therapeutic strategies for T2D.

## Introduction

Type 2 diabetes (T2D) is the most common type of diabetes and is a consequence of both downregulated insulin secretion and increased insulin resistance. T2D prevalence is increasing rapidly worldwide, and approximately 5 million people die from T2D-associated cardiovascular disease, cancers, and other liver and kidney diseases every year [1]. Both genetic and environmental factors contribute to T2D development. Large-scale, genome-wide association studies have identified genetic loci associated with T2D; however, genetic risk variants explain less than 20% of T2D heritability [2-4]. Indeed, environmental factors such as diet, exercise, age, and the gut microbiome contribute to the etiology of T2D because these aspects can alter the epigenetic landscape that lead to changes in chromatin structure and gene expression [5]. Epigenetic modifications are reversible yet heritable, and they do not result in an altered DNA sequence. The main epigenetic mechanisms are DNA methylation, histone modifications, and the action of noncoding RNAs and hence may mediate the effects of environmental factors on T2D.

DNA methylation is the best-studied epigenetic mechanism, entailing the addition of a methyl group to cytosine—mostly in the context of CpG dinucleotides. [6]. DNA methylation can suppress gene expression by interacting with histone deacetylases or interfering with transcription-factor binding [7]. DNA methylation plays an important role in development and disease because of its potential to alter gene expression. Therefore, the analysis of the DNA methylome at single-base resolution is a promising means for determining human disease etiology [8]. In this regard, both whole-genome bisulfite sequencing and Infinium methylation microarrays are efficient high-throughput methods for analyzing the methylation status of human DNA [9].

Genome-wide DNA methylation analysis has provided information concerning T2D-associated changes in DNA methylation. However, it has been argued that methylation changes are simply a secondary event that occurs at the chromatin level during disease progression [10]. This review posits that DNA methylation is both a possible cause and consequence of T2D. The first section introduces the role of metabolism in DNA methylation and the second section summarizes DNA methylation changes that drive T2D development.

## Metabolism Regulates DNA Methylation

Metabolism plays a central role in DNA methylation [11]. The methyl group required for DNA methylation is derived from S-adenosylmethionine [12], which is synthesized through the methionine cycle from several nutrients such as methionine, folate, choline, betaine and vitamins B2, B6, and B12 [12]. In rats, a diet deficient in S-adenosylmethionine leads to hypomethylation of liver DNA [13]. This dietary limitation affects not only global methylation but also the methylation of specific genes [14].

**Fig. 1.**
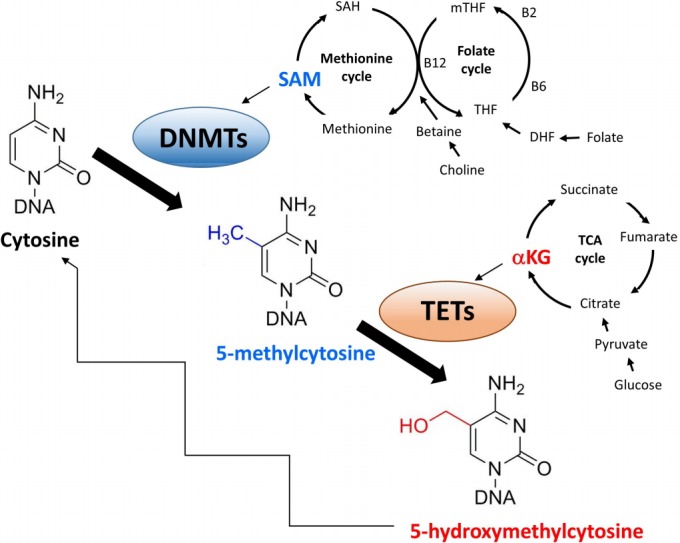
The role of metabolism in DNA methylation. DNA methyltransferases (DNMTs) catalyze the transfer of methyl group derived from S-adenosylmethionine (SAM), which is synthesized through the methionine cycle from several nutrients. Ten-eleven translocations (TETs) utilize the TCA cycle intermediate α -ketoglutarate (αKG) to remove methyl group. B2, vitamin B2; B6, vitamin B6; B12, vitamin B12; DHF, dihydrofolate; mTHF, 5-methyltetrahydrofolate; SAH, S-adenosylhomocysteine; THF, tetrahydrofolate.

The rate of DNA demethylation is also affected by metabolic fluctuations. Demethylation is regulated by ten-eleven translocation (TET) family enzymes, which utilize the TCA cycle intermediate α-ketoglutarate to remove methyl groups [15]. Each of fumarate and succinate acts as a competitor of α-ketoglutarate to inhibit TET activity [16]. Therefore, the cellular metabolic environment regulates the activity of enzymes involved in the balance between DNA methylation and demethylation (Fig. 1).

Metabolic perturbations can lead to epigenetic changes of immune cells, which may contribute to altered immune-cell function in metabolic diseases. Epigenomic alterations in immune cells are frequently observed in obesity and T2D [17]. For example, methylation of the two genes *UBASH3B* and *TRIM3*, which help regulate T-cell and macrophage proliferation and function, leads to impaired immune function in obese subjects [18]. Macrophages exposed to excess saturated fatty acids *in vitro* were found to express higher levels of DNA methyltransferase (Dnmt) 3B, resulting in enhanced M1 polarization and adipose-tissue inflammation [19]. Alterations in DNA methylation accumulate during the course of immune-system remodeling that occurs with metabolic disease, and therefore monitoring DNA methylation changes in immune cells could be a useful means of detecting metabolic complications [20].

## DNA Methylation Changes That Drive T2D Development

It has been suggested that accumulated errors in DNA methylation lead to altered gene expression, which can affect the response to external stimuli and contribute to T2D development [21]. Emerging data show that epigenetics plays a key role in the pathogenesis of T2D [22-24]. Genome-wide studies have identified altered DNA methylation patterns in pancreatic islets, adipose tissue, liver, and skeletal muscle from subjects with T2D compared with tissues of nondiabetic controls. In this section, I describe DNA methylation changes that may drive T2D development.

The pancreatic islets of Langerhans play central roles in the development of T2D. Blood glucose level increases after a meal, which triggers the secretion of insulin from pancreatic islet β-cells into the circulation; this is a fundamental means of controlling glucose homeostasis. β-cell failure impairs glucose tolerance and results in T2D [25]. Volkmar et al. [26] found that aberrantly methylated genes in T2D islets are associated with β-cell dysfunction and apoptosis. Genes for which DNA methylation (and therefore expression) is altered in human T2D islets (such as *CDKN1A, PDE7B*, and *SEPT9*) contribute to the perturbation of insulin and glucagon secretion [27]. Genome-wide analysis of DNA-methylation quantitative trait loci revealed that DNA methylation at single nucleotide polymorphism–CpG pairs in human islets underlies the observed genetic associations that affect gene expression. Functional studies revealed that genes such as *GPX7, GSTT1*, and *SNX19* directly affect β-cell proliferation and apoptosis, among other important biological processes [28].

Adipose tissue plays a central role in regulating whole-body energy metabolism. Adipose tissue stores energy in the form of lipids and acts as an endocrine organ that produces adipokines that control energy intake and energy consumption by other tissues [29]. In T2D, the dysregulation of normal adipose-tissue function leads to elevated levels of circulating lipids and increased lipid storage in alternative tissues such the liver, muscle, and pancreas [30]. Genome-wide DNA methylation analysis of subcutaneous adipose tissue of monozygotic twins discordant for T2D revealed that *CIDEC, CDKN2B, DUSP9, HNF4A, KCNQ1, TSPAN8*, and *VGLL1* were differentially methylated between the twins [31]. You et al. [32] demonstrated that overexpression of *Dnmt3a* is both necessary and sufficient for insulin resistance in adipocytes derived from mice or humans. These researchers also found that adipose-specific *Dnmt3a* knockout in mice protected the animals from diet-induced insulin resistance and glucose tolerance. They found that Dnmt3a mediates insulin resistance by methylating the *Fgf21* promoter; *FGF21* hypermethylation was evident in human subjects with T2D and correlated negatively with *FGF21* expression in human adipose tissue [32].

The liver is central for maintaining glucose homeostasis during both the fed and fasted states. During the fed state, insulin receptors on hepatocytes bind insulin, which induces glycogen synthesis/storage. During fasting, glucagon binding to hepatocytes leads to gluconeogenesis and glucose release [33]. This balance is lost in T2D, however, and insulin resistance in the liver contributes to hyperglycemia. Genes relevant to the development of T2D, such as *GRB10, ABCC3, MOGAT1*, and *PRDM16*, were found to be aberrantly methylated in the liver of T2D patients [33]. Interestingly, most of the liver-specific CpG sites in T2D patients are methylated to a lesser degree than in healthy controls. The hypomethylation found in the T2D liver may be explained by reduced folate levels in erythrocytes [33]. Kirchner et al. [34] reported decreased methylation of several genes controlling glucose metabolism within the ATF-motif regulatory site in the liver of severely obese nondiabetic and T2D patients, suggesting that obesity eventually leads to alterations of the liver epigenome, resulting in the upregulation of glycolysis and lipogenesis that may exacerbate insulin resistance. Metformin is the most common drug for treating T2D. Metformin decreases the DNA methylation of metformin transporter genes (*SLC22A1, SLC22A3*, and *SLC47A1*) in the human liver, thereby countering the increased methylation of these genes seen for T2D patients with hyperglycemia and obesity [35]. Abderrahmani *et al*. [36] found that decreased methylation and increased expression of *PDGFA* are associated with increased risk of both T2D and steatohepatitis.

Skeletal muscle is the primary site of insulin-induced glucose uptake and defects in skeletal-muscle metabolism contribute to insulin resistance [37]. Barres et al. [38] found increased methylation of genes involved in mitochondrial function, such as *PPARγ* and *PGC-1α*, using whole-genome promoter methylation analysis of skeletal muscle from normal subjects (i.e., glucose tolerant) and T2D patients. They provided evidence suggesting that *PGC-1α* methylation controls *PGC-1α* expression, a finding that is consistent with the reduced number of mitochondria found in cells of T2D patients; this links DNMT3B to the acute, fatty acid–induced, non-CpG methylation of the *PGC-1α* promoter [39]. Alibegovic et al. [40] observed increased methylation of *PPARGC1A* for T2D patients on bed rest, suggesting that physical inactivity promotes the establishment and maintenance of epigenetic marks that may increase T2D risk [40]. In a study of skeletal muscle of people with a family history of T2D, Nitert et al. [41] found differentially methylated DNA in genes of certain pathways, including the mitogen-activated protein kinase (MAPK), insulin, and calcium signaling pathways (e.g., *MAPK1, MYO18B, HOXC6*, and *PRKAB1*). Results from a DNA methylation analysis of skeletal muscle from healthy men before and after insulin exposure revealed increased *DAPK3* methylation, which is reduced in T2D patients [42]. Insulin and glucose modulate skeletal-muscle *DAPK3* methylation reciprocally, suggesting that a feedback mechanism controls *DAPK3* expression. [42]. Taken together, studies utilizing genome-wide methylation analysis of T2D patients as well as functional validation of target genes have identified many previously unknown DNA methylation changes that may promote T2D development.

Aging can be described as a time-dependent decline in multiple biological functions, such as a decline in resting metabolism and a reduction in epigenome stability [43]. In humans, age-related changes in DNA methylation patterns have been documented in blood, liver, brain, skeletal muscle, adipose tissue, and pancreatic islets [44]. Moreover, aging is associated with impaired pancreatic islet function [45] and therefore is a primary risk factor for T2D [46]. In general, the age of global epigenetic marks in certain genes correlates with upregulation of both the proinflammatory and interferon pathways and downregulation of the basal transcriptional machinery, DNA-damage response, and mitochondrial signatures [47]. Given that the number of older individuals is increasing dramatically worldwide and that aging is the greatest risk factor for the majority of chronic diseases including T2D, it is critical to understand the molecular-genetic basis of how aging promotes the development of chronic diseases and to develop novel multi-disease preventative and therapeutic approaches [48].

## Conclusion

**Fig. 2.**
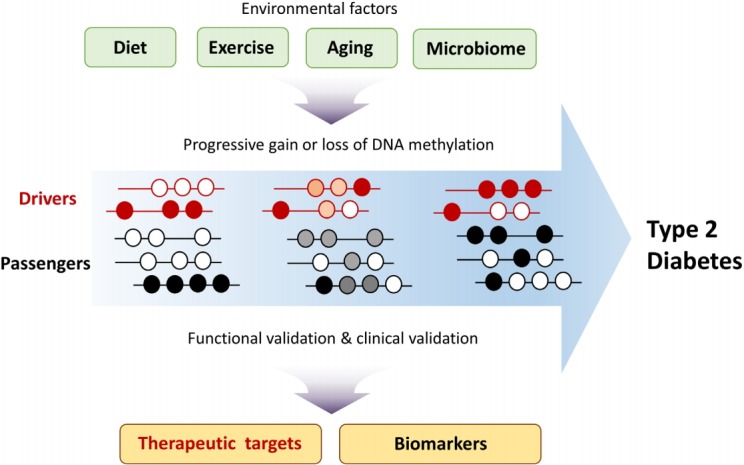
Environmental factors contribute to type 2 diabetes (T2D) development through epigenetic mechanisms including DNA methylation. Changes in driver methylation lead to changes in driver gene expression. Knowledge of DNA methylation changes can inform the development of biomarkers for T2D and enhance the prediction of T2D risk.

Dissecting the molecular mechanisms of T2D development is required for developing appropriate therapeutic strategies. Epigenome studies of T2D have identified T2D-specific changes in DNA methylation patterns. To determine whether such changes are drivers or passengers of T2D development, functional validation is needed [22]. In theory, driver DNA methylation changes should correlate with changes in the expression of T2D driver genes, and this correlation could inform the development of therapeutic drugs. Although changes in passenger DNA methylation are a consequence of T2D progression, such changes could be utilized as biomarkers for predicting T2D risk after clinical validation (Fig. 2). In addition to promoter regions, distal gene-regulatory elements such as enhancers and insulators should also be included when considering the potential impact of driver methylation changes on T2D. Analysis of long-range chromatin interactions using chromosome conformation capture–based techniques could elucidate the role, if any, that DNA methylation changes in distal regulatory elements play in T2D. The contribution of methylation at specific CpG sites to T2D development could be validated by targeted editing of DNA methylation sites using the CRISPR dCas9-Dnmt3a/Tet1 system [49,50]. Owing to the cellular heterogeneity of pancreatic islets, liver, and adipose tissue, T2D-specific DNA methylation changes may vary widely among different cell types. Recent advances in technologies such as single-cell transcriptomics and epigenomics could potentially enhance our knowledge of T2D development and its complications.
